# Prevalence of diabetes mellitus and impaired glucose tolerance in the urban population aged 30-69 years in Ribeirão Preto (São Paulo), Brazil

**DOI:** 10.1590/S1516-31802003000600002

**Published:** 2003-11-06

**Authors:** Maria Teresa da Costa Gonçalves Torquato, Renan Magalhães Montenegro, Luis Atílio Losi Viana, Rui Augusto Hudari Gonçalves de Souza, Carla Márcia Moreira Lanna, Júlio César Batista Lucas, Cláudio Bidurin, Milton Cesar Foss

**Keywords:** Diabetes mellitus, Glucose intolerance, Prevalence, Brazil, Diabetes mellitus, Intolerância a glicose, Prevalência, Brasil

## Abstract

**CONTEXT:**

Diabetes mellitus prevalence has been increasing worldwide due to factors like lifestyle changes and higher life expectancy. The Brazilian Multicenter Study performed between 1986 and 1988 evaluated the prevalence of diabetes and impaired glucose tolerance.

**OBJECTIVE:**

To assess the prevalence of diabetes and impaired glucose tolerance in the urban population aged 30-69 years of the city of Ribeirão Preto, SP, Brazil.

**TYPE OF STUDY:**

A two-stage, cross-sectional home survey.

**SETTING:**

Ribeirão Preto, São Paulo, Brazil.

**PARTICIPANTS:**

A random sample of 1,473 individuals.

**METHODS:**

The sample plan was drawn up using a sampling scheme of stage conglomerates according to sex, age and family head income. Subjects were first screened by fasting capillary glycemia (FCG). Those that screened positive (FCG 100 mg/dl) and every seventh consecutive person who screened negative (FCG < 100 mg/dl) was submitted to a 75 g oral glucose tolerance test. The diagnosis of diabetes and impaired glucose tolerance were based on World Health Organization criteria.

**RESULTS:**

The overall rates of diabetes and impaired glucose tolerance were 12.1 and 7.7%, respectively. Men and women had similar rates of diabetes (12.0 vs. 12.1%) and impaired glucose tolerance (7.9 vs. 7.3%). Differences in the rates for whites (11.6%) and nonwhites (13.3%) for diabetes were not significant, while impaired glucose tolerance was more prevalent among whites. The prevalences of diabetes and impaired glucose tolerance ranged from 3.3% and 2.6% in the 30-39 year age group to 21.7% and 11.3% in the 60-69 year age group, respectively. Obese subjects (BMI ≥ 30 kg/m^2^) and those with a family history of diabetes (first-degree relatives) presented higher prevalences of diabetes (22.6% and 19.7%, respectively).

**CONCLUSIONS:**

The prevalence of diabetes in Ribeirão Preto was found to be comparable to that occurring in developed countries. With respect to the Brazilian Multicenter Study we verified an increased prevalence of diabetes but a similar prevalence of impaired glucose tolerance. These findings may reflect modifications in environmental factors and lifestyle that have been occurring in Brazilian cities like Ribeirão Preto, especially regarding increasing rates of sedentary living and obesity .

## RESUMO

**CONTEXTO:**

A prevalência de diabetes mellitus tem aumentado, em decorrência das mudanças no estilo de vida e do aumento da expectativa de vida da população. . No Brasil, o estudo que avaliou as prevalências de diabetes e intolerância a glicose foi o Estudo Multicêntrico Brasileiro, realizado de 1986 a 1988.

**OBJETIVO:**

Determinar a prevalência de diabetes e intolerância a glicose na população urbana, de 30 a 69 anos, do município de Ribeirão Preto (SP), Brasil.

**TIPO DE ESTUDO:**

Estudo transversal com inquérito domiciliar em duas etapas.

**LOCAL:**

Ribeirão Preto, São Paulo, Brasil.

**PARTICIPANTES:**

Amostra randomizada de 1.473 indivíduos.

**MÉTODOS:**

O planejamento amostral adotado foi de conglomerados em estágios considerando sexo, faixa etária e renda do chefe dos domicílios Os indivíduos eram rastreados com glicemia capilar de jejum (GCJ). Aqueles com resultado positivo (glicemia capilar em jejum ≥ 100 mg/dl) e a cada sétimo com resultado negativo (< 100 mg/dl) eram submetidos a sobrecarga oral com 75 g de glicose. Os diagnósticos de diabetes e intolerância a glicose foram baseados nos critérios da Organização Mundial da Saúde.

**RESULTADOS:**

As prevalências de diabetes e de intolerância a glicose foram de 12,1 e de 7,7%, respectivamente. Homens e mulheres apresentaram freqüências semelhantes de diabetes (12,0 versus 12,1%) e intolerância a glicose (7,9 versus 7,3%). Diferenças entre brancos (11,6%) e não-brancos (13,3%) para diabetes não foram significantes, enquanto a intolerância a glicose foi mais prevalente em brancos . As prevalências de diabetes e intolerância a glicose variaram de 3,3% e 2,6%, no grupo de 30-39 anos, a 21,7% e 11,3% no grupo de 60-69 anos, respectivamente. Indivíduos obesos (índice de massa corpórea ≥ 30 kg/m^2^) e aqueles com história familiar de diabetes (parentes de primeiro grau) apresentaram prevalência maior de diabetes: 22,6% e 19,7%, respectivamente.

**CONCLUSÕES:**

A prevalência de diabetes em Ribeirão Preto se mostrou comparável à observada em países desenvolvidos. Em relação ao Estudo Multicêntrico Brasileiro, verificamos maior prevalência de diabetes, mas prevalência semelhante de intolerância a glicose. Estes achados podem refletir o impacto dos fatores ambientais e de estilo de vida, que têm ocorrido em cidades brasileiras como Ribeirão Preto, principalmente nas taxas crescentes de sedentarismo e obesidade.

## INTRODUCTION

Over the last decade, diabetes mellitus has emerged as an important clinical and public health problem throughout the world. In the United States, 8 million adults have been diagnosed with diabetes mellitus and 90 to 95% of them have type 2 diabetes. The prevalences of known cases of diabetes mellitus are 6 to 7% for persons aged 45 to 64 years, and 10 to 12% for those aged 65 years and older. In addition to the known cases, it has been estimated that 8 million persons fulfill the diagnostic criteria for diabetes mellitus but remain undiagnosed. Approximately 35 to 45 million adults have impaired glucose tolerance, which is a condition known to present high risk of progression to diabetes mellitus.^1^ According to the "Diabetes Health Economic Study Group" of the International Diabetes Federation (IDF), it is expected that there will be 300 million people with diabetes mellitus throughout the world in 2025.^2^

In 2000, the prevalence of diagnosed diabetes among US adults was 7.3%, the prevalence of obesity (body mass index ≥30 kg/ m^2^) was 19.8% and the prevalence of both combined was 2.9%.^3^

The prevalence of diabetes mellitus in Brazil is comparable with US and European data. In the Brazilian Multicenter Study,^4^ the prevalences of diabetes mellitus and impaired glucose tolerance were 7.6 and 7.8%, respectively. This study was conducted on a representative sample of the urban Brazilian population aged 30 to 69 years in nine Brazilian capitals between 1986 and 1988, and was the first population-based survey in Brazil that used World Health Organization criteria for the diagnosis of diabetes,^5^ thus allowing international comparisons. No other studies of this type have been conducted in Brazil since then.

Over recent decades, the prevalence of diabetes mellitus has been increasing worldwide due to factors like changes in lifestyle (sedentary living and eating habits) and obesity.^1,2,3,4^ These are perhaps a consequence of urbanization and modernization of cities, and higher life expectancy for the general population and diabetic subjects, probably caused by the overall improvement in healthcare.

Diabetes is considered to be an important cause of morbidity and mortality for different age groups. According to the World Health Organization report, cardiovascular disease appears as the second biggest cause of death worldwide. Type 2 diabetes is the tip of an iceberg of cardiovascular disease risk factors that also includes hypertension, dyslipidemia and obesity (metabolic syndrome). The natural consequence from this will be an epidemic of coronary heart disease and stroke, as well as the microvascular complications of diabetes.^6^ An analytical study of a population sample of 546 type 2 diabetics from Ribeirão Preto (São Paulo) has demonstrated higher frequency of macrovascular, microvascular and neurological complications in patients with poor metabolic control and a longer course of diabetes.^7^ Similar data had already been obtained for type 1 diabetics from the same area.^8^ Recently, in the United Kingdom Prospective Diabetes Study (UKPDS), it was demonstrated that improvement in glycemic control reduces macrovascular and microvascular complications in type 2 diabetes.^9^ Equivalent results had been already demonstrated in the Diabetes Control and Complication Trial (DCCT), in relation to type 1 diabetes.^10^

The coefficient of prevalence for a disease reflects its magnitude, and its determination is important since it provides a basis for the planning of health actions related to that disease. Thus, with this objective, the present study was delineated to carry out an epidemiological survey to evaluate the prevalence of diabetes mellitus and impaired glucose tolerance in a representative sample of the population of Ribeirão Preto (SP), Brazil.

## METHODS

This study was a cross-sectional home survey started in September 1996 and concluded in November 1997 in Ribeirão Preto, a medium size city in the state of São Paulo, located in the southeastern region of Brazil. A random sample of 1.473 subjects aged 30 to 69 years, excluding pregnant women, was studied using a methodology similar to that applied in the Brazilian Multicenter Study.^4^

The sample plan was drawn up using a sampling scheme of stage conglomerates. For the initial composition of the study population, the 1991 demographic census data from the Brazilian Institute of Geography and Statistics (IBGE) was utilized.^11^ As its first factor, the sample plan took into account sex, age (from 30 to 69 years) and distribution of the population according to the census sections. For its second factor, it utilized the income of heads of families (in numbers of minimum wages) in permanent private homes, due to the imprecision of this variable. For the definition of the conglomerates, the study used preexisting geographical divisions of the city already used by the City Health Department of Ribeirão Preto, called "Regional Health Areas", which include the Primary Care and District Health Units.

Starting from these divisions, we mapped each of these areas as a function of the census sections. Each conglomerate was characterized on the basis of the sum of the census sections corresponding to each regional area, according to age, sex, and family head income. Based on these sums, we calculated what each category of variables represented in relation to the whole regional area. Once the conglomerates were defined, we selected at random census sections in each conglomerate and then homes. After section distribution, we made a random selection of 20% of the census sections (20% of the sections in each regional area). Thus, we surveyed 7,856 subjects between 30 and 69 years of age in 5,005 homes (Table 1).

**Table 1:** Selected sample of the population of the city of Ribeirão Preto that participated in the second phase of the study (screening for diabetes and oral glucose tolerance tests), according to age group, sex, skin color, educational level and family history of diabetes in first-degree relatives

				Screened sample for diabetes			75 g oral glucose tolerance test sample	
	Population of Ribeirão Preto	eligible	interviewed	total	positive	negative	screened positive	screened negative
Participants (n)	173,190	7,856	1,633	1,473	469	1,004	363	137
Age groups (%) (years)								
30-39	40.6	39.4	24.7	24.0	12.4	29.4	13.8	27.7
40-49	27.4	28.2	26.0	25.8	23.0	27.1	25.3	22.6
50-59	19.0	19.8	26.5	26.8	34.1	23.4	35.6	25.6
60-69	13.0	12.6	22.8	23.4	30.5	20.1	25.3	24.1
Sex (%)								
Male	47.3	48.7	34.3	33.5	36.5	32.1	36.9	29.9
Female	52.7	51.3	65.7	66.5	63.5	67.9	63.1	70.1
Skin color (%)	-	-						
White	-	-	83.3	83.0	85.7	81.7	86.9	79.6
Nonwhite	-	-	16.7	17.0	14.3	18.3	13.1	20.4
Education (%)	-	-						
Basic	-	-	71.5	71.8	75.7	70.0	74.3	72.0
Secondary and university	-	-	28.5	28.2	24.3	30.0	25.7	28.0
Diabetes in the family (%)	-	-						
Yes	-	-	45.9	46.0	51.2	43.6	47.0	39.4
No	-	-	54.1	54.0	48.8	56.4	53.0	60.6
First-degree relative with diabetes (%)								
Yes	-	-		34.5	41.6	31.2		
No	-	-		65.5	58.4	68.8		

To calculate the number of homes to be visited, we initially determined the minimum sample size, considering an expected prevalence of 7.6% based on the Brazilian Multicenter Study data,^4^ and a confidence interval of 95%, with a maximum error of 2% in estimating the prevalence proportion. We then selected homes in each census section by drawing lots. The local population was informed and encouraged to participate through the media.

The survey was carried out by medical students of the Faculdade de Medicina de Ribeirão Preto – Universidade de São Paulo, trained and directly supervised by senior endocrinologists of the City Health Department of Ribeirão Preto.

The survey consisted of two phases. First, the homes were visited and the population received detailed information about diabetes mellitus and the purpose and importance of the study. Then, men and non-pregnant women between 30 and 69 years of age were invited to participate in the study. Those who agreed to participate answered a simple and objective questionnaire for the identification of known cases of diabetes and risk factors for diabetes mellitus and cardiovascular disease. They were informed about the second phase of the study and the need for a 12 to 14 hour fast.

In the second phase of the study, a screening test consisting of fasting capillary glycemia (FCG) determination was performed on the following morning. The subjects with glycemic levels ≥ 100 mg/dl (5.6 mmol/l) and < 200 mg/dl (11.1 mmol/l) were immediately submitted to a 75 g oral glucose tolerance test with determination of capillary glycemia after 120 minutes. Every seventh consecutive individual who screened negative (FCG < 100 mg/dl) was also submitted to the glucose load test.

The diagnosis of diabetes mellitus and impaired glucose tolerance was based on World Health Organization criteria.5 Previously-diagnosed individuals and those with fasting or two-hour capillary glycemia ≥ 200 mg/dl (11.1 mmol/l) were considered to be diabetics. Subjects with two-hour capillary glycemia ≥ 140 and < 200 mg/dl (≥ 7.7 and < 11.1 mmol/l) were considered to have decreased glucose tolerance; and those with FCG < 100 mg/dl (5.6 mmol/l) or two-hour capillary glycemia < 140 mg/dl (7.7 mmol/l) were considered to have normal glucose tolerance. Glycemia was determined by using glucose oxidase reagent strips with immediate reading via portable reflectance meters (Advantage, Boehringer-Mannheim, USA).

Prevalences were adjusted for age by a direct method, using as its standard the population of Ribeirão Preto from the 1991 IBGE demographic census. The χ^2^ test was used for analyzing the representativeness between samples and comparing the prevalence rates. The level of significance was set at 5%.

## RESULTS

There were 7,856 individuals from 5,005 homes, ranging in age from 30 to 69 years, in the four areas selected for the study. A total of 1,333 permanent private homes in 17 census sections previously picked by drawing lots were visited.

A total of 1,633 subjects were enrolled but only 1,625 satisfied the inclusion criteria of the study; 1,546 of them (96%) were included in the first phase of the study and the remaining subjects (4%) were not at home at the time of the interview or refused to participate. A total of 1,473 (90.6%) individuals were included in the second phase of the study, thus forming the database group for the analysis of diabetes and impaired glucose tolerance prevalence (Table 1).

Of the 1,473 individuals, 1,004 showed fasting glycemia < 100 mg/dl (screened negative) and of these, 137 (the sum of every seventh individual that was screened negative) were submitted to the 75 g oral glucose tolerance test. Of the 469 individuals with fasting glycemia ≥ 100 mg/dl (screened positive), 363 (those without fasting glycemia ≥ 200 mg/dl or with previously diagnosed diabetes) were also submitted to the 75 g glucose tolerance test (Table 1).

According to the World Health Organization criteria,^5^ of the 1,473 subjects tested, 1,181 (80.2%) were classified as individuals with normal glucose tolerance, 114 (7.7%) as having impaired glucose tolerance, and 178 (12.1%) as diabetics. Women and men had similar rates of diabetes mellitus and impaired glucose tolerance (12.1% and 12% for diabetes mellitus and 7.9% and 7.3% for impaired glucose tolerance, respectively). When this rate of diabetes (12.1%) was applied to the Ribeirão Preto population projections for 2003, the number of people aged 30-69 years with a diagnosis of diabetes was estimated at 27,739.

By evaluating raw and age-adjusted rates, we were able to observe a statistically significant progressive increase in the prevalences of diabetes mellitus and impaired glucose tolerance according to age subdivided into decades, ranging from 3.3% and 2.6% in the 30-39 year age group to 21.7% and 11.3% in the 60-69 year age group, respectively. It can be noted that in the latter group, the prevalences of diabetes mellitus and impaired glucose tolerance were 6.5 and 4.3 times higher then in the former group, respectively (Table 2).

**Table 2:** Prevalence of diabetes and impaired glucose tolerance according to age group and sex in Ribeirão Preto

	Women				Men				Total			
	Diabetes		Impaired glucose tolerance		Diabetes		Impaired glucose tolerance		Diabetes		Impaired glucose tolerance	
	Raw data	Age adjusted	Raw data	Age adjusted	Raw data	Age adjusted	Raw data	Age adjusted	Raw data	Age adjusted	Raw data	Age adjusted
Age group (years)	*	*	*	*	*	*	*	*	*	*	*	*
30-39	2.6	3.0	3.0	3.5	3.4	3.9	0.8	0.9	2.8	3.3	2.2	2.6
40-49	6.4	6.4	6.0	6.0	8.5	8.5	7.7	7.7	7.1	7.1	6.6	6.6
50-59	15.1	13.5	11.4	10.2	15.4	13.9	6.5	5.8	15.2	13.6	9.8	8.8
60-69	25.1	23.1	11.2	10.3	20.6	19.2	14.0	13.0	23.5	21.7	12.2	11.3
**Total**	**12.1**	**12.1**	**7.9**	**7.9**	**12.0**	**12.0**	**7.3**	**7.3**	**12.1**	**12.1**	**7.7**	**7.7**

* *Statistical differences between age groups in the same column (p < 0.05).*

Of the 178 diabetic subjects, 134 (75%) had been previously diagnosed and 44 (25%) were detected in the study. In the latter group, five (11.4%) had FCG ≥ 200 mg/dl (11.1 mmol/l) and 39 (88.6%) were diagnosed by the 75 g oral glucose tolerance test. Of these, 36 (81.8%) showed fasting glycemia between 100 mg/dl (5.6 mmol/l) and 199 mg/dl (11.0 mmol/l) and three (6.8%) had fasting glycemia of < 100 mg/dl (5.6 mmol/l). Therefore, the prevalences of previously diagnosed diabetic subjects and new cases detected in this study were 9.3% and 2.8%, respectively. Of the 114 individuals classified as having impaired glucose tolerance, 96 (84.2%) screened positive (FCG ≥ 100 mg/dl) and 18 (15.8%) screened negative (FCG < 100 mg/dl) and were also submitted to the 75 g oral glucose tolerance test (every seventh consecutive subject). The prevalences of previously known and newly diagnosed cases of diabetes by age group and sex are presented in Table 3. There was no difference in diabetes prevalence between men and women. In the previously diagnosed group, a statistically significant progressive increase in prevalence was observed, according to age group distribution.

**Table 3:** Prevalence of previously known and newly diagnosed diabetes according to age group and sex in Ribeirão Preto

	Women				Men				Total			
	Previously known		Newly diagnosed		Previously known		Newly diagnosed		Previously known		Newly diagnosed	
	Raw data	Age adjusted	Raw data	Age adjusted	Raw data	Age adjusted	Raw data	Age adjusted	Raw data	Age adjusted	Raw data	Age adjusted
Age group (years)	*	*			*	*			*	*		
30-39	2.6	3.0	-	-	2.5	2.9	0.9	1.0	2.5	2.9	0.3	0.4
40-49	4.0	4.0	2.4	2.4	5.4	5.4	3.1	3.1	4.5	4.5	2.6	2.6
50-59	11.1	9.9	4.0	3.6	10.5	9.5	4.9	4.4	10.9	9.8	4.3	3.8
60-69	20.2	1B.6	4.9	4.5	16.5	15.4	4.1	3.8	1B.9	17.5	4.6	4.2
**Total**	**9.3**	**9.3**	**2.8**	**2.8**	**8.7**	**8.7**	**3.3**	**3.3**	**9.1**	**9.1**	**3.0**	**3.0**

* *Statistical differences between age groups in the same column (p < 0.05).*

Of the 134 previously diagnosed cases, 5 (4%) were not under treatment, 25 (19%) were being treated by diet alone, 87 (64%) were taking oral anti-diabetic drugs, 12 (9%) were on insulin therapy, and 5 (4%) were taking oral anti-diabetic drugs in combination with insulin therapy.

Using the FCG, we classified the previously diagnosed diabetic subjects as under good (< 120 mg/dl or < 6.7 mmol/l), regular ( ≥ 120 and < 140 mg/dl or ≥ 6.7 and < 7.8 mmol/l) and poor control (≥ 140 mg/dl or ≥ 7.8 mmol/l). Forty-eight individuals (36%) were found to be under good control, 23 (17%) under regular control and 63 (47%) under poor control.

In Table 4, the prevalence of diabetes mellitus is presented according to skin color (self-reported), educational level, family history of diabetes, and presence of obesity (body mass index ≥ 30 kg/m^2^). Differences in the rates of diabetes mellitus between whites (11.6%) and nonwhites (13.3%) were not statistically significant, while impaired glucose tolerance was more prevalent among whites (8.3%) than in nonwhites (4.8%).

**Table 4:** Prevalence of diabetes and impaired glucose tolerance in the city of Ribeirão Preto according to skin color, educational level, family history of diabetes in first-degree relatives and presence of obesity (BMI ≥ 30 kg/m^2^)

		Diabetes		Impaired glucose tolerance
	Previously known	Newly diagnosed	Total	Total
Skin color (%)				
White	8.7	2.8	11.6	8.3*
Nonwhite	9.7	3.6	13.3	4.8
Educational level (%)				
Basic	9.5*	3.4*	12.9*	8.3*
Secondary and university	4.9	1.5	6.4	6.2
Diabetes in the family (%)				
Yes	13.3*	3.1	16.4*	6.3
No	5.0	3.0	8.0	9.1*
First-degree relative with diabetes (%)				
Yes	16.1*	3.5	19.7*	7.1
No	5.4	2.7	8.1	8.1
Obesity (%) (BMI ≥ 30 kg/m^2^)				
Yes	15.6*	7.0*	22.6*	13.4*
No	6.8	1.9	8.7	6.3

* *p < 0.05; BMI = body mass index.*

Individuals with only primary school education (basic) presented twice (12.9%) the prevalence of diabetes mellitus as those with secondary school education and university education (6.2%). The prevalence of impaired glucose tolerance was 8.3% in the first group and 6.2% in each of the other two groups.

Considering first-degree relatives, subjects with positive and negative family histories of diabetes presented diabetes mellitus prevalences of 19.7% and 8.1%, respectively, and similar prevalences of impaired glucose tolerance. In previously diagnosed diabetics the prevalence of diabetes mellitus was 3 times higher (16.1%) in subjects with a positive family history (5.4%), while in the group diagnosed in the study there was no significant difference between groups, i.e. subjects with a positive and negative family history presented diabetes mellitus prevalences of 3.5% and 2.7%, respectively. When any family history of diabetes was considered, individuals with a positive history showed double the prevalence (16.4%) of subjects with no relatives with diabetes mellitus (8%).

Obese subjects (body mass ≥ index 30 kg/m^2^) presented 2.5-times higher prevalence of diabetes mellitus (22.6%) and 2.1-times higher prevalence of impaired glucose tolerance (13.4%) than for non-obese individuals (8.7% for diabetes mellitus and 6.3% for impaired glucose tolerance). Among previously and newly diagnosed diabetics, the prevalences of diabetes mellitus were 15.6% and 7% in obese subjects and 6.8% and 1.9% in non-obese subjects, respectively.

## DISCUSSION

In the present study, the prevalence of diabetes mellitus in Ribeirão Preto was 12.1%. This is comparable to what has been reported for developed countries. According to the National Health and Nutrition Examination Survey (NHANES III, 1988-1994), the prevalence of diabetes mellitus in the U.S. population when World Health Organization criteria^5^ were applied was 14.3% in subjects aged 40-74 years.^12^ In comparison with the Brazilian Multicenter Study performed between 1986 and 1988, the last study in Brazil that evaluated the prevalence of diabetes mellitus (mean of 7.6%),^4^ we observed an increased rate.

The prevalence of diabetes mellitus has been increasing worldwide, particularly in industrialized and urban populations of developing countries. Corroborating such a relationship, it was seen in the Brazilian Multicenter Study (1987) that the city of São Paulo, one of the most urbanized and industrialized centers in Brazil, and also the capital of the state where Ribeirão Preto is located, presented the highest prevalence of diabetes mellitus (9.7%).^4^ This phenomenon may explain the elevated prevalence found in Ribeirão Preto, since this city has experienced growing urbanization and modernization over recent decades. The similar prevalence of impaired glucose tolerance observed in this study (7.7%), in relation to the Brazilian Multicenter Study (mean of 7.8%),^4^ supports this hypothesis. It may also suggest that while modifications in environmental factors have taken place, especially in terms of increasing rates of sedentary living and obesity as a consequence of lifestyle changes, the genetic background has remained unaffected. However, it needs to be taken into account that we have used the mean Brazilian prevalence of diabetes mellitus for comparison. In other words, there are no local parameters to be applied for comparison, since this is the first study of diabetes mellitus and impaired glucose tolerance prevalence performed in Ribeirão Preto. Considering the multi-ethnicity and the social and economic characteristics of our population, and that this urbanization process is taking place in several areas of the country, it is possible that this higher prevalence of diabetes mellitus found in Ribeirão Preto reflects growth in the prevalence of this disease that may be occurring in Brazil as a whole.

The concept that aging, heredity, and obesity are universal risk factors for type 2 diabetes is well known.^13^ Among these factors, positive family history of diabetes represented the third highest risk factor for diabetes mellitus in our study, with overall prevalence of 16.4% and prevalence of 19.7% when considering only first-degree relatives. These rates are higher than the mean Brazilian prevalence found in the Brazilian Multicenter Study^4^ conducted 10 years ago, in which the prevalence of diabetes among individuals with positive family history of diabetes mellitus was 12.5%.

Conversely, subjects with positive and negative family histories of diabetes presented similar prevalence of impaired glucose tolerance in Ribeirão Preto, when first-degree relatives were considered. This suggests that heredity is not as important in impaired glucose tolerance as in diabetes mellitus. These results are in accordance with a previous cross-sectional study of 21,057 individuals in Västerbotten (Sweden) conducted by Lindahl et al.^14^ In this, the distribution of impaired glucose tolerance and its relationship with age, obesity and hereditary background of diabetes were evaluated. It was found that more than 70% of subjects with impaired glucose tolerance had no hereditary background of diabetes.

In the Brazilian Multicenter Study,^4^ age was the factor that determined the highest risk for diabetes mellitus, with 17.4% prevalence in the 60-69 year-old group. In the city of Rio de Janeiro, Brazil, diabetes mellitus prevalence was 22.4% among the women of this same age group.^15^ In Ribeirão Preto, this factor was the second most important, although with an even higher prevalence of diabetes mellitus (21.6%) in the same age group. Obesity also increased the prevalence of diabetes mellitus, which was 22.6% in our study, in comparison with 10.3% in the Brazilian Multicenter Study. This represented the main risk factor for diabetes mellitus when compared with a positive family history of diabetes and aging. Although all of these factors probably contribute towards explaining the increased prevalence of diabetes mellitus found in our study, it may be suggested that the increasing prevalence of obesity is the major factor responsible for this.

A substantial increase in the prevalence of obesity in the United States has been shown in the Hispanic Health and Nutrition Examination Survey (HANES).^16^ Obesity has important health, social and economic consequences for the community. Its increase worldwide has had a significant impact on the global incidence of cardiovascular disease, type 2 diabetes, cancer, osteoarthritis, work disability and sleep apnea. It has also been postulated that disability due to obesity-related type 2 diabetes is set to increase, particularly in industrializing countries.^17^

Tuomilehto et al.^18^ reported on the effects of changes in lifestyle on the development of type 2 diabetes in a trial involving subjects with impaired glucose tolerance. During this trial, the risk of diabetes was reduced by 58% among overweight subjects with impaired glucose tolerance, by reducing weight and dietary saturated fat and increasing fiber intake and physical activity.^18^

Thus, obesity prevention programs should be on the scientific and political agenda not only in developed but also in developing countries. In our city, there are multi-professional teams acting in six different regions and a regular physical activity program is available at primary health care units, working towards the prevention of cardiovascular risk factors.

In the present study, diabetes had similar prevalence in whites and nonwhites. Our results are in agreement with what was observed in the Brazilian Multicenter Study of 1987.^4^ In contrast, in the United States population the prevalence of diabetes mellitus among non-Hispanic blacks and Mexican-Americans was 1.6 to 1.9 times higher than among non-Hispanic whites.^16^ However, the miscegenation of the population of Ribeirão Preto, which is similar to what is found in the overall Brazilian population, needs to be taken into account. This impairs comparisons with other countries.

Diabetes and impaired glucose tolerance were more prevalent among people of lower educational level in our city. They were, respectively, 2.0 and 1.3 times higher among subjects with only basic education. These differences may be related to the increased occurrence of other risk factors in this population such as obesity. The prevalence of obesity evaluated in the same population was higher among people of lower educational level (unpublished data). These differences were not found in the earlier Brazilian Multicenter Study^4^ when averages for the country as a whole were considered, although similar results were found in specific regions.

In this study, 25% of the diabetics ignored their condition, compared with 50% in the Brazilian Multicenter Study,^4^ and only 4% of the previously diagnosed subjects had not received any treatment, compared with 22% in the previous study. This improvement was probably the consequence of information campaigns and healthcare improvements. Screening for gestational diabetes has been included in routine prenatal care, and diabetes detection and treatment programs have been implemented locally and nationally over the last few years. Medications for continuous use in diabetes, hypertension and hypercholesterolemia treatment are being supplied to the population of our municipal district.

Diabetes mellitus is considered to be a major public health problem. It is important to determine the prevalence of a disease, because this gives support for the planning of health policy. Therefore, in addition to quantifying the occurrence of diabetes mellitus, the determination of its prevalence in our region has allowed health actions to be better managed. This knowledge has provided guidelines for health policy concerning diabetics and risks within the population (unpublished), and has allowed calculations of what resources are needed for medications, outpatient and hospital care. It has enabled awareness among the population and health professionals regarding this disease to be raised, thereby reducing the frequency of diabetes complications and, consequently, reducing public health costs. Health education for the population is fundamental in improving the primary prevention of type 2 diabetes, in order to stimulate changes in lifestyle, especially in terms of the control of obesity and physical inactivity.

## CONCLUSIONS

In the present study, the prevalence of diabetes mellitus in Ribeirão Preto was found to be comparable to that occurring in developed countries. In relation to the Brazilian Multicenter Study, we have observed increased prevalence of diabetes mellitus but similar prevalence of impaired glucose tolerance. These findings may reflect the modifications in environmental factors that have been taking place in Brazilian cities undergoing progressive urbanization, such as Ribeirão Preto, especially with regard to increasing rates of sedentary living and obesity as a consequence of lifestyle changes.

## APPENDIX

The following members of the Diabetes Association of the Centro Acadêmico Rocha Lima, Faculdade de Medicina da Universidade de São Paulo, Ribeirão Preto campus, participated in this study: Abenor M. Minaré Filho, Alessandro H. Brunetti, Alexandre H. Macchetti, Alexandre P. Goulart, André Armani, André M. Wada, Anelise Bettarelo, Antônio L. Lins Neto, Carina T. Souza, Carla M. de Souza, Carlos R. R. Marcondes, Carolina W. Aloisi, Claúdia O. Baraldi, Daniela T. Melo, Danilo B. Massone, Eduardo B. Oliveira, Eduardo C. Dornelas, Ernandes K. Nakamura, Flávio V. Papa, Fábio H. Mendonça, Fábio Kawakami, Fábio Zenha, Fabrício L. M. da Silva, Flávia R. V. Dalmonte, Gabriel S. Vasconcelos, Irineu Renzi Jr., José Carlos Coutinho, José Eduardo F. Passos, José Maurício Dias Jr., Juliana V. Rossi, Juliana B. Barra, Kátia H. Uejma, Kleber S. E. Santo, Luciano H. Melato, Luis Fernando Joaquim, Marcela B. Magalhães, Marcelo B. Silva, Maria Lúcia L. Abib, Mônica A.C. Teixeira, Murilo M. Coelho, Pablo F. Uchoa, Paulo T.C. Prado, Rafael D.S. Coelho, Rafael Nita, Ralph S. Dibbern, Renata A. Feracini, Ricardo S. Oliveira, Rony P. Lopes, Rui L. Feitosa Jr., Ruth H. de Morais, Sandra R. Tsukada, Sinichiro Maeda, Tarcila C.C. Santos, Tarciso Schimbeck, Tatiane G. Cabral, Viviane P. Tietz.
